# Species distribution models: A comparison of statistical approaches for livestock and disease epidemics

**DOI:** 10.1371/journal.pone.0183626

**Published:** 2017-08-24

**Authors:** Tracey Hollings, Andrew Robinson, Mary van Andel, Chris Jewell, Mark Burgman

**Affiliations:** 1 Centre of Excellence for Biosecurity Risk Analysis, University of Melbourne, Melbourne, Australia; 2 Ministry for Primary Industries, Wellington, New Zealand; 3 Department of Health and Medicine, Lancaster University, Lancaster, United Kingdom; 4 Centre for Environmental Policy, Imperial College, London, United Kingdom; University of New England, AUSTRALIA

## Abstract

In livestock industries, reliable up-to-date spatial distribution and abundance records for animals and farms are critical for governments to manage and respond to risks. Yet few, if any, countries can afford to maintain comprehensive, up-to-date agricultural census data. Statistical modelling can be used as a proxy for such data but comparative modelling studies have rarely been undertaken for livestock populations. Widespread species, including livestock, can be difficult to model effectively due to complex spatial distributions that do not respond predictably to environmental gradients. We assessed three machine learning species distribution models (SDM) for their capacity to estimate national-level farm animal population numbers within property boundaries: boosted regression trees (BRT), random forests (RF) and K-nearest neighbour (K-NN). The models were built from a commercial livestock database and environmental and socio-economic predictor data for New Zealand. We used two spatial data stratifications to test (i) support for decision making in an emergency response situation, and (ii) the ability for the models to predict to new geographic regions. The performance of the three model types varied substantially, but the best performing models showed very high accuracy. BRTs had the best performance overall, but RF performed equally well or better in many simulations; RFs were superior at predicting livestock numbers for all but very large commercial farms. K-NN performed poorly relative to both RF and BRT in all simulations. The predictions of both multi species and single species models for farms and within hypothetical quarantine zones were very close to observed data. These models are generally applicable for livestock estimation with broad applications in disease risk modelling, biosecurity, policy and planning.

## Introduction

Policy and planning for the livestock sector is often impeded by the lack of reliable records on spatial distributions and abundance of livestock species [1]. Few countries maintain accurate up-to-date livestock census data, particularly at the resolution and spatial scale required [1, 2], hampered by high costs and considerable ongoing resource requirements. Yet livestock demographic information is critical in environmental, social and epidemiological applications, and especially for disease risk modelling and emergency response [1, 3].

Rising demand for animal products is leading to the global expansion and intensification of livestock industries [2]. A concomitant increase in global movement of animals and animal products via international trade is providing greater chances for the transmission of invasive pests and diseases into naïve countries [4], which can have catastrophic economic, social and animal welfare impacts. For example, the eight-month foot-and-mouth disease (FMD) outbreak in the United Kingdom in 2001 led to the culling of more than six million animals for disease control and ‘welfare’ [5], and cost the UK more than £7 billon [6], including substantial restrictions on trade and reductions in tourism. Availability of a complete census of vulnerable animals would facilitate a rapid response for the containment and eradication of an incursion such as FMD, and therefore minimise the impacts.

Statistical modelling can be used to fill gaps where data are too expensive and/or onerous to collect. Species distribution models (SDM) predict species distributions by relating occurrence or abundance records to environmental, demographic, climatic and satellite derived predictor variables [7]. Greater availability of candidate predictor data have resulted in the rapid rise of SDMs in ecological and conservation applications (e.g., [7]), and more recently for agriculture (e.g., [3, 8, 9]) and disease risk modelling (e.g., [10, 11]). The ecological and environmental requirements of most livestock species are well understood, and relevant training and predictor data are often readily available, both of which make widespread predictions feasible.

To date, models that predict livestock and commercial poultry populations have focused almost exclusively on stratified regression [2, 3, 8, 12–14]. A recent exception assessed the use of Random Forests to improve estimates [9]. Reasonable predictive accuracy (correlations of up to 80% between predicted and direct counts) has been demonstrated at local, regional [2, 12] and global scales [1]. In most of these studies training data has comprised the disaggregation of coarse spatial resolution census data into animal densities. For example, the Gridded Livestock of the World project (GLW) estimated global distributions at 1km^2^ resolution for major livestock species of cattle, goats, buffalos, sheep, pigs and poultry [3]. The GLW data has been applied in animal health research [15] including for predicting the risk of avian influenza [16] and the incidence and distribution of FMD [17].

Machine learning methods have been proposed for livestock estimation due to their potentially higher predictive performance, and their capacity to straightforwardly incorporate complex interaction effects and noisy data [3, 7]. Advances in computing capacity, software and statistical innovation [18] have made machine learning techniques such as Random Forests (RF), boosted regression trees (BRT) and k-nearest neighbour (K-NN) models, practical options for large ecological datasets [3]. Methods such as RF and BRT are relatively novel for ecological applications but they consistently outperform more established methods [7, 19].

Studies evaluating SDM techniques have arisen in response to the large variety of models available, types of data and research questions (e.g., [7, 20–22]), as well as the considerable variation in predictive performance between SDM types [7]. Models for common, widespread and generalist species generally are less accurate than environmentally or geographically restricted species [7, 21, 22]. Therefore, examining and comparing machine learning techniques and SDM approaches that use livestock data is critical to ensure the appropriate models are applied, given their growing popularity, and the potential value and application of the predictions.

New Zealand has one of the strongest biosecurity frameworks in the world [23]. Its geographic isolation and strict biosecurity regulations have prevented the incursion of many significant agricultural pests and diseases which is crucial to maintaining trade access to international markets, and to protecting the economy and industry. Biosecurity investment by the NZ government is substantial but the recent and economically damaging incursions of varroa mite [24] and bacterial kiwifruit vines disease (PSA) [25] show that significant agricultural pests still pass the border undetected. New Zealand has an accurate, up-to-date national-level database of the geographic locations of farms called Agribase; however counts of livestock on these holdings are not reliably available. Yet, this information is important for biosecurity and disease preparedness [26, 27], highlighted by the recent estimated cost of a small FMD outbreak in NZ involving several hundred farms to be more than NZ$16 billion over eight years [28].

We developed and tested three machine learning species distribution models to predict national-level farm animal demographic data: BRT, RF and K-NN. The models were built from a commercial livestock database, Agribase, and environmental and socio-economic predictor data for New Zealand. The context of the model development was to (i) provide support for decision making in an emergency response situation and (ii) initial values for stochastic models that are used for planning. We assessed their performance using a regional cross-validation with model predictions evaluated for local government areas to assess how well models can predict to new regions. The models were assessed at two spatial scales: (i) at the farm level, and (ii) using hypothetical quarantine zones whereby model predictions were assessed in a 3km radius around randomly selected ‘outbreak’ farms reflecting the emergency quarantine zones that may be established immediately following notification of a serious disease such as FMD.

## Methods

### Livestock data

AgriBase^™^ (AsureQuality) is a national-level commercial livestock database that holds contact data and geospatial information about agricultural and horticultural properties across NZ. The database was initially developed for biosecurity and emergency management applications and currently lists 134,377 individual properties in vector polygon data format. Entry in the database is voluntary and more than 60% of the properties have records for livestock. ‘Lifestyle farms’, small holdings with few animals used primarily for personal consumption, on the urban fringe are the most numerous farming type by a factor of almost four (>60,000), followed by commercial beef (15,433) and dairy farms (11,840). No data on enterprise activities of any kind are held for 36,815 properties, of which 87% are categorised as ‘lifestyle’ farms; the majority of these are considered to be missing data rather than true zeroes. This is due to the voluntary nature of the database with no incentive for property owners to declare their livestock holdings. However, because it was not possible to distinguish between missing values and values that are really zero, all were removed for model development and assessment. There were also approximately 6,000 farms with identical spatial information reflecting cross-leased land where information from multiple polygons has been merged into a single data point. This prevented the correct extraction of predictor information for the individual farm polygons and therefore precluded their use in the models. In total 91,662 properties were used for model training and validation. It was not possible to differentiate between free-range and intensively farmed animals.

Many livestock demographic applications require knowledge of the total numbers of livestock contained on a property. We built models with a consistent response variable across properties by calculating the livestock units (LSU) as grazing equivalents for each property. This value was calculated by multiplying the total number of animals of a given species by a value that represents their grazing equivalence to an adult dairy cow (Table 1; [29, 30]). In certain contexts knowledge of specific livestock species is required, so single species models were also assessed for cattle, as derived from beef and dairy records.

**Table 1:** Species equivalence values to an adult dairy cow that were used for analysis [29, 30].

Livestock unit (LSU) coefficients	LSU
Dairy cows	1.00
Beef cows	0.80
Deer	0.80
Horses	0.80
Pigs	0.40
Goats	0.10
Sheep	0.10
Poultry (not used in this study)	0.01

### Predictor variables

Predictor variables were calculated and/or extracted for individual property polygons from spatial data layers using the open-source statistical environment, R version 3.1.1 (libraries: 'rgeos', 'rgdal', 'maptools', 'raster', 'geoshpere', and 'sp'; [31–37]). The 22 candidate predictor variables that could potentially influence livestock distributions and farming types were a combination of environmental (slope, aspect, elevation, pasture quality, distance to river), climatic (mean annual rainfall, mean annual temperature) and demographic (social deprivation index, distance to major road, distance to urban centre which partly accounts for human population density) variables (S1 Table). Variables were chosen based on the availability of datasets (e.g., [38, 39]) and factors that may be relevant for both livestock presence and abundance. Environmental and climatic factors account for natural species limitations and tolerance thresholds, for example, food and water availability, effects of aspect on vegetation and solar radiation, and temperature. Demographic variables account for human factors associated with livestock production, including market access and human population density [3, 12].

The total absolute area of each property was divided into four separate predictor variables: high quality pasture, low quality pasture, forest, and ‘other’ land cover calculated as the remaining area of the property polygon, all measured in hectares [39].

In the models both the standard deviation and the mean of several variables were used to account for variability across individual properties e.g. some properties may be very hilly, but just using the mean of the slope would not account for valleys and peaks.

### Spatial stratifications

Models were built and evaluated with training and validation data based on regional stratification, using randomly selected local government authority areas, to assess the ability of the models to predict to new regions. NZ mainland has 16 different regional councils, hereafter called regions (S1 Fig). Training data comprised a stratified sample of 11 randomly selected regions, six from the north island (out of nine) and five from the south island (out of seven) and the validation data comprised the remaining 5 withheld regions. Two hundred independent training and validation datasets were generated in this way. This analysis also provided information on whether prediction accuracy of the models differed across regions, reflecting different environmental and demographic conditions.

Model performance was assessed at two spatial scales. The first was a farm-level comparison of the predictions and the actual livestock counts in the withheld regions. The second scale assessed the adequacy of the models in emergency disease response situations by validating model predictions aggregated to nominal “quarantine zones” around randomly selected farms. We chose a radius of 3 km to reflect the emergency response quarantine zones that typically would be established immediately following notification of a serious disease such as FMD. A grid of 9000m x 9000m cells was overlaid across NZ, providing approximately 3,000 grid cells. The property intersecting with the centroid of each grid cell was extracted using ArcGIS (Version 10.2.2). The cell size was chosen to minimise overlap of farms within 3km of the central property, if two cells were randomly selected next to each other. Two hundred of these intersecting properties were selected at random and then each was used as the hypothetical ‘outbreak farm’, the focal point for a 3km quarantine zone. All farms within a 3km radius of the focal property boundary were extracted and used as validation data using the spatial libraries ‘rgeos’, ‘rgdal’, and ‘sp’ in R [33, 35–37]. The 200 ‘outbreak farms’ provided a balance between providing enough validation data for modelling without overlapping zones becoming an issue. Ten iterations of these methods provided ten independent grids, to give a total of 2000 outbreak farms and concomitant quarantine zones (200 outbreak farms x 10 independent datasets). For each new iteration the bottom left corner of the grid was moved at random to produce ten distinct grids, and thereby different sets of grid centroids to intersect with farms. Since sampling of the outbreak farms was performed spatially by proximity to the farm centroid, this sample was taken with probability proportional to the outbreak farm size. The remaining farms outside the 200 hypothetical quarantine zones were used as training data.

### Models tested

Three SDM modelling techniques were considered. The optimal parameters were assessed for each model type prior to fitting the final models and are outlined for each model type below. Each model strategy assumes that the sample is representative of the population, a common statistical assumption. A formal interpretation of our assessment of the models requires the assumption that the residuals are uncorrelated; this is unlikely because of the spatial nature of the data; however, such assumptions are again common and we do not think it substantially undermines our conclusions.

#### Random forests (RF)

RF is a bagging algorithm that creates decision trees by repeatedly selecting bootstrap samples from a training set, fitting trees to each replicate. Bagging randomly generates a set of data from the original with replacement. A random sub-sample of the predictors is used to split each node in a tree [40]. The given number of trees is averaged to obtain the estimates. Fitting variables can be changed in the model, including the number of trees to be grown and the number of randomly selected variables to be used as candidates at each split. RFs were built using the ‘randomForest’ package in R [37, 41] with the number of trees set to 2000. For the RF models, each response variable was transformed using a square root, based on statistical practice (the metrics are counts, for which the square root is a common variance-stabilizing transformation) and empirical inspection of residual plots.

#### Boosted regression trees (BRT)

BRT’s combine two algorithms, namely regression trees and boosting, to build and fit many models and improve predictions by focusing resources on outliers. The algorithm builds a large number of simple decision trees adaptively. The final BRT model can be thought of as an additive regression model [42]. Several fitting variables can be varied in the model: a user-defined bagging fraction introduces stochasticity into the model and defines the proportion of data drawn at random from the original data at each step; the learning rate varies the contribution of each tree added to the model and the tree complexity defines the number of nodes for each tree [42]. Predictive performance was assessed under different bagging conditions, learning rate and tree complexity and the parameters were chosen based on those which achieved the minimum predictive error. Parameters in the final BRT models had a bagging factor of 0.5, learning rate of 0.005 and tree complexity of 10. Boosted regression trees (BRT) were implemented using the ‘dismo’ and ‘gbm’ packages in R [43, 44]. For the BRT, the response variable was assumed to have a conditional Poisson response and log link function.

#### K-nearest neighbour (K-NN)

K-NN is a distance-weighted nearest neighbour prediction algorithm in which training sites closest in predictor distance to the new point of interest are used to predict its value. Closer training samples can be weighted higher than those further away. The output of the k-NN for regression is the average value of the *k* nearest neighbours, where the *k* nearest properties were used to predict the LSU/cattle of the target properties. We defined the number of samples (*k*) as 5, and the distance metric used was Euclidean distance. K-NN models were fit using the ‘class’ package in R [45]. No transformation was needed for the K-NN models because they make no assumption about the conditional distribution of the response variable.

### Model development and evaluation

The predictive accuracies of Boosted regression trees (BRT), Random forests (RF) and K-nearest neighbour (K-NN) were evaluated and compared for national-level livestock estimation in NZ. Two response variables were separately modelled to test multi-species aggregates and single species models: LSU rounded up to the nearest whole number to give counts of LSU, and cattle as a single species model case study, derived from beef and dairy records. For each response variable, 200 replicate BRT, RF and K-NN models were run for the two spatial stratifications, corresponding to the ten independent training and validation datasets. The models were set up with the same 22 predictor variables (S1 Table). The models were assessed for how well they predicted livestock on individual farms and the aggregated farms within quarantine zones.

The model fits were determined primarily by how well they predicted LSU in the withheld samples. A goodness of fit measure between the withheld validation data and predicted values was used to compare the BRT, RF and K-NN models: the discrepancy quantified using the Root Mean Square Prediction Error (RMSPE).

$$RMPSE_{i}=\sqrt{\sum_{i=1}^{n}{\left({\hat{y}}_{i}-y_{i}\right)}^{2}}$$

where the quantity was only computed for those farms in the regions that were excluded from the replicate sample, and ${\hat{y}}_{i}$ was computed by predicting the farm (or summing the predicted farms to the quarantine zone) LSU or cattle.

Pseudo R^2^ was also calculated as the relative variance of the observed values minus the predicted values from withheld observations, against the variance of the observed values, averaged across all repetitions.

## Results

### Methods comparison

Boosted regression trees were the best performing models overall when assessed on RMSPE and Pseudo R^2^ (Table 2). However the prediction accuracy of random forests was higher for many regions (Table 2, S2 and S3 Tables). This was the case for farm-level and quarantine zone predictions for both LSU and cattle. When assessing the RMSPE by farm size, overall RF predictions were substantially better than BRTs for all but very large farms (> 250 LSU) (Table 3). Random forests predicted better for farms with zero values and farms with less than 15 LSU, which is common for over 90% of lifestyle farms. All models had very high R^2^ values for zone level predictions (LSU: BRT = 0.92; RF = 0.91; KNN = 0.74), substantially higher than for farm level predictions (Table 2). These differences in goodness of fit measures between RF and BRTs mean the choice of model should be based on context of model development and requirements of the model predictions.

**Table 2:** The prediction root mean squared error (RMSPE) and Pseudo R^2^ for LSU and cattle using the withheld results for the regional spatial stratification for individual farms within regions and quarantine zones. Also shown is the mean count of LSU and cattle per farm and the standard deviation in brackets. Full results for all models are shown in the Supporting Information (S2 and S3 Tables).

Response variable	Spatial stratification	Mean (SD)	RMSPE			Pseudo R^2^		
			RF	BRT	KNN	RF	BRT	KNN
**LSU**	**Farm**	131.5 (342.1)	215.6 (45.4)	216.4 (48.2)	380.2 (57.3)	0.60	0.59	-0.28
	**Zone (‘000)**	12.7 (19.6)	5.4 (1.0)	4.8 (0.8)	9.0 (1.2)	0.91	0.92	0.74
**Cattle**	**Farm**	89.9 (237.2)	172.9 (31.8)	171.4 (31.6)	275.3 (32.4)	0.47	0.47	-0.39
	**Zone (‘000)**	7.8 (14.7)	6.4 (1.3)	3.5 (0.6)	5.2 (1.4)	0.86	0.92	0.75

**Table 3:** Comparison of BRT, RF, and KNN by the RMSPE for LSU across different size farms.

Farm Size (number of LSU)	Number of farms	RF	BRT	KNN
**No animals**	16650	61.5	74.4	292.0
**Small (1–15)**	34582	31.0	37.3	111.6
**Medium (16–150)**	18623	82.3	99.8	252.3
**Large (151–250)**	5618	134.9	152.7	312.8
**Very large (>250)**	15919	505.4	498.5	780.5

Both BRT and RF performed significantly better than K-NN across all spatial stratifications and response variables (Table 2, S2 and S3 Tables). No single K-NN model performed better than BRT and RF with any goodness of fit measure, with the same predictor and response data. K-NN predictions included zero values, but overall predictions were poor.

Number of farms per quarantine zone was 64 on average, and the average by region ranged from 25.5 to 101. The mean number of zones per region was 125, and the range was 2 to 445.

**Fig 1:**
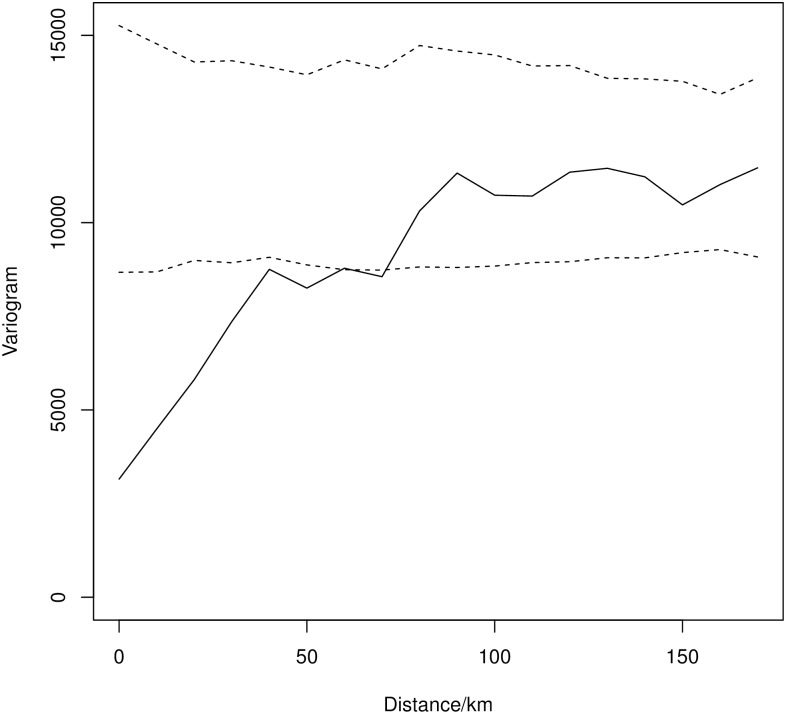
Spatial variogram of farm-level RMSPE, using Euclidean distance between farm centroids. The solid line gives the variogram of the observed data, with the dotted lines indicating the 95% confidence intervals of the null distribution obtained by random permutation of RMSPE between the farm locations.

There was significant residual spatial correlation of farm-level RMSPE below approximately 60km, with farms close together tending to have similar LSU values than farms further apart (Fig 1).

**Fig 2:**
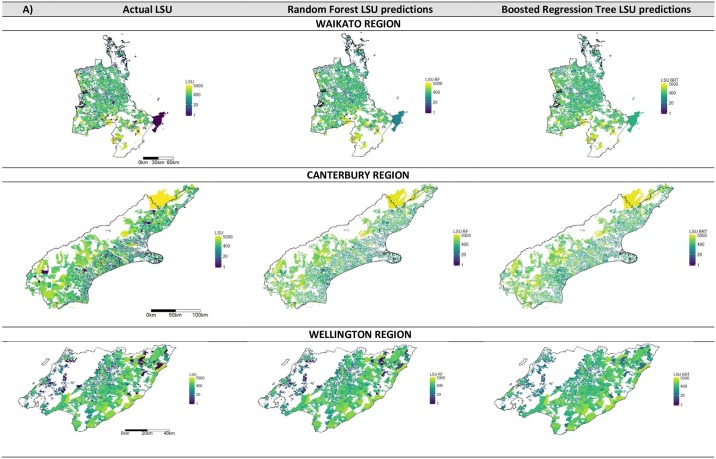
Maps of the actual livestock units (LSU) and the modelled region level livestock predictions for three regions. Maps show results from Random Forests and Boosted Regression Trees for Waikato, Canterbury and Wellington for region predictions. The gradient colour axis is on a log scale for display purposes. KNN results are not shown due the extremely poor fit of the models relative to other model types.

**Fig 3:**
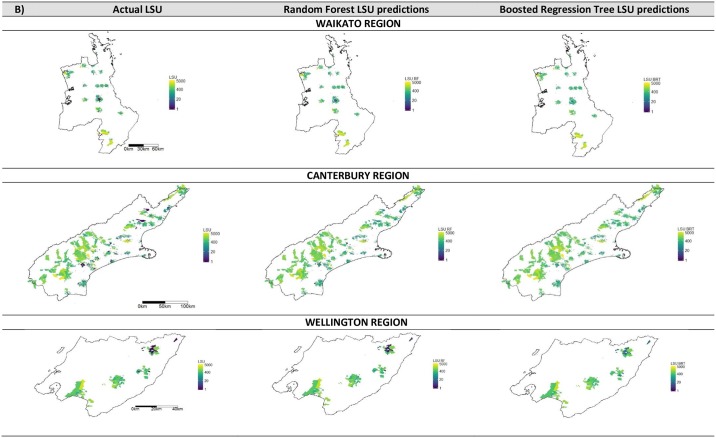
Maps of the actual livestock units (LSU) and the modelled zone level livestock predictions for three regions. Maps show results from Random Forests and Boosted Regression Trees for Waikato, Canterbury and Wellington for zone level predictions. The gradient colour axis is on a log scale for display purposes.

Examples of three regions are shown spatially in Figs 2 and 3 for the actual LSU, and the predicted LSU from both Random Forests and Boosted Regression Trees for the regional stratification (Fig 2) and quarantine zone stratification (Fig 3). KNN is not shown due to its extremely poor model fit.

### Region cross-validation with random forests

#### Farm level predictions

**Fig 4:**
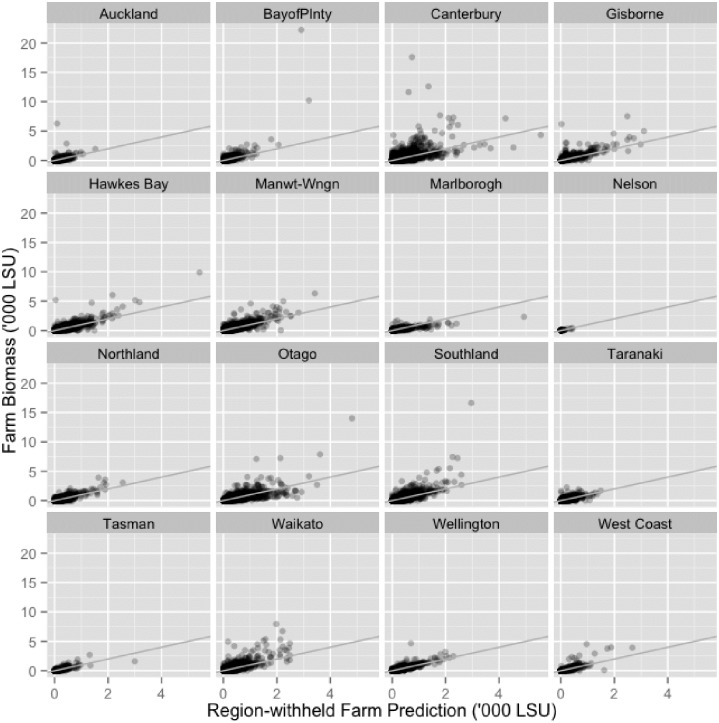
Scatterplot of the observed against the predicted farm-level. Results are plotted by region. Total quarantine zone LSU was calculated by summing the observed and predicted values for all farms within the 3km quarantine zone. Cattle results are not shown as plots they are a subset of LSU.

Fig 4 provides a scatterplot of the observed against the fitted farm-level LSU values for the region-withheld RF model, by region. The results for all regions tended to cluster closely around the 1:1 line, which confirmed the model quality. The predictions also showed evidence of fanning, meaning that the natural variation increases with the size of the response variable. The fanning was accommodated in the model by means of a square-root transformation of the response variable, as noted in the Methods. The scatterplots of cattle results are not shown, as they are a subset of the LSU variable and relationships are similar to LSU across all region simulations.

#### Quarantine zone predictions

**Fig 5:**
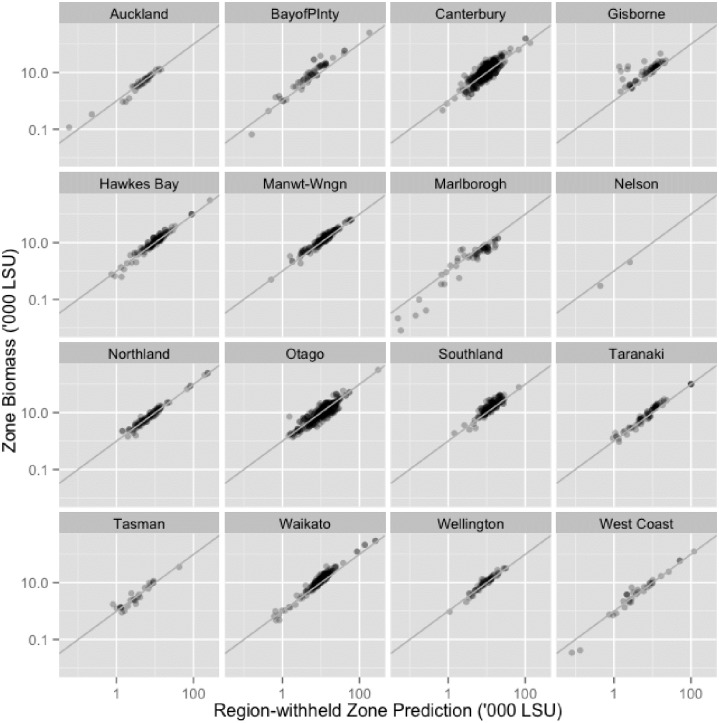
Scatterplot of the observed against the predicted total quarantine zone LSU. Results are plotted by region. Total quarantine zone LSU was calculated by summing the observed and predicted values for all farms within the 3km quarantine zone. Cattle results are not shown as plots they are a subset of LSU.

The results for the quarantine level predictions for the regional stratification model are summarised in Table 2, S2 Table and Fig 5. We summed the predictions for every farm that had any area within a 3 km radius of the centroid of the subject farm, and applied the same statistics to these summed quarantine-level predictions and observations. Again, the results clustered closely around the 1:1 line, and as expected there was much less variation around the line than in farm-level predictions.

### Variable importance

We determined the most important variables for predicting both LSU and cattle by running the random forest model on the full dataset with all covariates, including a categorical spatial predictor variable representing regional councils (S1 Fig). Comparing separate models for each of the two response variables can identify differences in variable importance for multi species and single species models.

**Fig 6:**
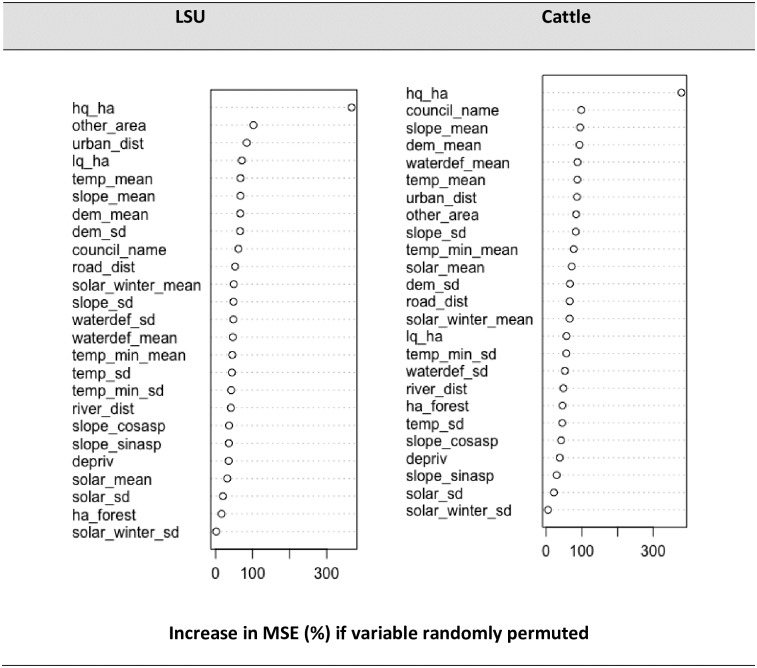
Relative influence of predictor variables from random forest models for LSU and cattle. Plots show the increase in MSE of predictions if the variable of interest were randomly permuted. See S1 Table for full variable names and descriptions.

Fig 6 shows the relative influence of predictor variables for the RF models for both LSU and cattle response variables. The x axis represents the mean decrease in accuracy, as measured by an increase in mean square error (MSE), if the variable were randomly permuted. High quality pasture was overwhelmingly the most important predictor for both LSU (367%) and cattle (378%) and had substantially more influence than the second highest ranked predictor variable. For LSU the size of the farm, accounted for by the combination of area, high quality pasture, low quality pasture and the variable ‘other area’ was very important, as was the distance to an urban centre. For cattle, the region, as well as variables accounting for the slope and altitude of the farm were the most highly ranked variables after high quality pasture. Variables accounting for aspect (slope cosine and slope sine), deprivation index and the two solar standard deviation predictor variables were not important for either response but still had some impact on model performance, given by positive relative importance measures.

## Discussion

Comparative species modelling exercises illuminate the strengths and weaknesses of different techniques [46] but have not previously been undertaken for livestock data, and machine learning techniques have been applied to livestock data only very recently [9]. The three machine learning techniques assessed in this study, RF, BRT and K-NN, produced vastly different results even with identical predictor and response variables, as seen in other ecological studies (e.g., [7, 21]). Overall predictive accuracy of the best models at the zone level were extremely high, though with a tendency to underestimate total numbers, possibly due to zero-inflation. Predictions for farm-level livestock in withheld regions were lower, and models for LSU generally performed better than for cattle alone.

Discrepancies existed between the goodness of fit measure for RF and BRTs for some simulations and regions which make it difficult to determine a single best model. Boosted regression trees were the best performing technique overall, but RF’s performed equally well or better in many simulations. Random forests performed best for all but very large, typically commercial farms, which make them more valuable in disease response modelling and national livestock estimation, where data deficiencies tend to be clustered around smaller non-commercial and peri-urban farms. RMSPE was used as an indication of model accuracy [3] and it is heavily penalised for large departures from observed values. The poor fit of RF for large farms relative to BRT may mean the RMSPE for RF were disproportionately influenced relative to BRT. K-NN models performed poorly relative to the other models across all simulations.

For NZ, the context of the spatial model development was to provide support for decision-making in an emergency response situation and in initialising planning models, and therefore RF’s are recommended given their better performance for farm sizes of most interest. The demonstration of residual spatial correlation in the results indicates the need for methodological developments, combining model-based spatial correlation methods [47] with non-parametric machine learning techniques. For areas consisting largely of commercial grazing (e.g. parts of the south island) BRT’s could be considered. Stochasticity of populations at a local level means that model predictions should be used with caution for disease control decisions during an outbreak. Epidemic responses could be severely impacted by missing a piggery in the outbreak zone. The predictions however are valuable for regional and national responses to diseases, as well as planning and simulation modelling.

Widespread species, such as livestock, generally have complex distributions and often do not respond predictably to environmental gradients. Such species are expected to be better modelled with non-parametric models such as RF, BRTs and K-NN [21]. Random forests [19] and BRTs [7] are relatively novel in ecology though they have been widespread in other disciplines for some time. These machine learning techniques require greater computing capacity than classical tools such as generalized linear models, but are generally considered to have great predictive accuracy [3, 7, 19]. Interaction terms are often omitted from traditional SDM techniques because they greatly increase the number of model parameters [18], but RF and BRT can easily fit complex relationships and interactions without the need to explicitly specify them, they do not overfit and are stable with noisy data [7, 19, 42]. BRT’s have successfully been used for disease and disease risk modelling (e.g., [16, 48]) and RF’s for the presence of rare and invasive plant species [19]. The capability of machine learning techniques, and RF in particular, for population mapping is being realised and many organisations are beginning to investigate these techniques including FAO and the WorldPop consortium [3, 9, 49]. Our results provide support for their use in agricultural applications.

RFs have some advantages over other machine learning techniques. They can compute the contribution of each predictor through post-hoc analysis of variable importance measures, even where interactions exist [19, 49], which has previously been cited as a challenge facing SDMs [50]. RF’s also have fewer user-defined parameters than BRT’s, which can be of significant benefit when using automated fitting procedures [49]. The final advantage of RF over KNN and BRT is technological: at present, it is relatively straightforward, although time consuming, to produce distributions for the point predictions, using quantile RF [51]. These distributions can then be used to represent and propagate the statistical uncertainty of the prediction through instances of model simulation, although care must be taken to consider the potential correlation of prediction errors.

For livestock predictions, area of high-quality pasture was the most important predictor for all model simulations (Fig 6). Farm slope, elevation and hilliness were also important and reflect what livestock species can be kept in certain areas, e.g. high country farms, renowned for steep terrain and winter snow, are often confined to sheep farming [52]. Incorporating socio-economic and anthropogenic variables can improve model performance [3] and this was demonstrated with the high relative importance of distance to urban centre and distance to road. This is likely to be the influence of lifestyle farms on the urban fringe. Similarly, human population density was a key predictor in determining livestock distributions in the GLW regression models [1], and large population centres and roads were both important in explaining cattle farm locations in Australia [26]. In our study, deprivation index, a measure of socio-economic status, was not statistically important for the LSU or cattle. Our results suggest that RF can be used successfully to estimate peri-urban livestock populations, despite other studies citing the potentially confounding issues of multifunctional land-use and human settlement [13]. In other studies, areas of lowest animal density on the periphery have been more difficult to accurately model with higher coefficients of variation and levels of uncertainty relative to higher density areas [12].

Different farm types and livestock species can play varying roles in disease transmission depending on context [53–55]. In the case of FMD, infected cattle become infected easily and produce large quantities of virus relative to sheep, which can have limited pathology and act as silent spreaders [56, 57]. Cattle were used in this study as a case study to compare single species vs multispecies model predictions. Single species models can provide specific information about the species’ distribution and important predictor variables, demonstrated in the differences in ranking for important variables. For cattle, the regional identifier variable was very important, indicating that regional variations influence the distribution and abundance of livestock species. In NZ this is particularly the case for dairy, which is usually concentrated in specific areas and it may be worthwhile investigating the merits of further disaggregation into dairy and beef cattle. This outcome also indicates that spatial stratification is important. Similar work by Prosser et al [2], estimating poultry populations, found regionally stratified models performed better than country wide models on the basis that the factors driving livestock densities will be similar in comparable ecological and administrative areas [3].

The spatial sampling approach used to identify ‘outbreak’ farms for establishing quarantine zones selected the farms with probability proportional to their area. This would not be an issue in disease response modelling as the outbreak farm would be known, rather than randomly selected. Region is a more unbiased measure for choosing predictor data but also has biases related to environmental, geographic and other factors. For example, certain regions in the south island may comprise mainly large-scale sheep farms due to the hilly terrain and low productivity land, and these types of farms may not be adequately represented if this region were removed for model validation. Different model performance based on spatial stratification has been observed in other studies but with much larger scale differentials. Similar work by Prosser et al [2], estimating poultry populations, found regionally stratified models performed better than country wide models on the basis that the factors driving livestock densities will be similar in comparable ecological and administrative areas [3].

Unbiased data are rare for large numbers of species across extensive geographical areas [50] and low density areas can increase the uncertainty of predictions [2, 12]. This was true for the Agribase^™^ database used in this study. First, the database is voluntary which makes differentiating missing data from true zeroes all but impossible. The majority of the ‘missing’ data was attributed to small peri-urban lifestyle farms. However, the removal of this data was unlikely to have had significant effects on model predictions because one third of the remaining data was classified as lifestyle farms, suggesting they were well represented in the data including with respect to location and variability of livestock holdings. Second, discrepancies existed for some polygons, which were represented by merging of multiple farm ids and/or discontinuous polygons. All were removed due to the substantial investment required to investigate and separate each group of polygons relative to the likely cost to model accuracy. This data represented a small fraction of the database (<5%) and was not expected to introduce any significant biases. Additional errors were expected to arise from seasonal influences on animal movements and breeding, economic factors, droughts, etc. which can all affect the temporal and spatial representativeness of census data (Kao, 2002). None-the-less the model predictions had high accuracy, but true validation would require on the ground farm surveys [3].

Livestock estimation is most commonly represented as numbers per square area (e.g., [2, 3, 12, 13]). However, this representation is problematic for estimating numbers in individual grid cells because livestock populations are spatially and temporally dynamic [3]. This study estimated livestock for each property, which is arguably a more valuable measure by allowing incorporation of farm-level attributes, with greater utility for disease risk planning and policy applications. Two significant reasons demonstrated the importance of having this level of information during the 2001 FMD outbreak in the UK, in which decision makers relied heavily on models built on livestock census and location data. First, farm location data informed disease containment and eradication strategies (e.g. culling and vaccination) which relied on spatial relationships between susceptible host populations [53, 58]; second, farm-level attributes including size, livestock numbers, species mix and number of land parcels, are all significant risk factors in FMD transmission [53, 54]. This was the first time that mathematical modelling had been used for management of an outbreak [59].

The quantity and quality of livestock census data available for the 2001 FMD UK outbreak was unprecedented [55], but despite the acknowledged value, few countries maintain comprehensive agricultural census data and/or geospatial databases [58]. The resources required to collect and maintain databases can be prohibitive, particularly on large- or national-scales. The application of SDMs for livestock estimation could potentially be extrapolated to other countries where little or no data is available provided reasonable quality, high resolution data on environmental conditions and farming systems are available and comparable. Good livestock predictions have been obtained for countries where data is only available at low resolution [12] or comprehensive training data is scarce [26]. The machine learning techniques presented here may be better at predicting to new or under-sampled areas as they have greater ability to work with noisy data and incorporate complex interactions. A problem facing SDM techniques in the future for livestock applications is the intensification of farming practises which causes a disassociation between livestock and their environments, making it difficult to predict populations based on environmental attributes [3, 13]. This is already the case for chickens and pigs in many countries [3].

## Conclusion

Animal biosecurity threats will increase concomitantly with the demand for livestock products, which is projected to double in the next two decades [60]. Several recent high profile biosecurity outbreaks highlight the value of up-to-date census data. For managers to implement the most effective disease control strategies they need sufficient information to make informed decisions [23], and maintaining or establishing activities for continual preparedness enables a quick response [61]. The SDM approaches in this study are internationally applicable for livestock population estimation and have broad applications in disease research, biosecurity, as well as in policy and planning.

## Supporting information

S1 Table Summary of the source and calculation of predictor variables used for modelling. There are 22 predictor variables, with variation in some predictors across properties represented by both standard deviation and mean of the variable.
(DOCX)

S2 Table Model results for livestock units (LSU). The LSU prediction root mean squared error (RMSPE) using the withheld results for both individual farms within regions and to 3km quarantine zones. Table also shows the mean number of LSU per farm in the region, the standard deviation for all farms in brackets, and the total number of farms in each region. Results for Quarantine zone predictions are in 1,000s of LSU.
(DOCX)

S3 Table Model results for cattle. The cattle prediction root mean squared error (RMSPE) using the withheld results for both individual farms within regions and to 3km quarantine zones. Table also shows the mean number of cattle per farm in the region, the standard deviation for all farms in brackets, and the total number of farms in each region. Results for Quarantine zone predictions are in 1,000s of cattle.
(DOCX)

S1 Fig Map of livestock units (LSU) per hectare across New Zealand from Agribase^™^. White areas represent areas removed such as cross leased land or areas of missing data. Areas of more than 10 LSU/cattle per hectare are represented in dark green. LSU represented per hectare is used for display purposes only.
(TIF)
