# Supplementary material for: Species distribution models: A comparison of statistical approaches for livestock and disease epidemics
Source: PLoS One. 2017 Aug 24;12(8):e0183626. doi: 10.1371/journal.pone.0183626 (PMC5570337; doi:10.1371/journal.pone.0183626)
Supplement: S1 Table — There are 22 predictor variables, with variation in some predictors across properties represented by both standard deviation and mean of the variable. (DOCX) [file pone.0183626.s001.docx]

**S1 Table*:*** **Summary of the source and calculation of predictor variables used for modelling.** *There are 22 predictor variables, with variation in some predictors across properties represented by both standard deviation and mean of the variable.*

| **Variable (Abbreviation)** | **Description** | **Source of data** |
| --- | --- | --- |
| Distance to main roads (ROAD_DIST) | Calculated from the centroid of the farm property to the nearest major sealed road from vector line layer | Land and Information NZ (LINZ) https://data.linz.govt.nz/ |
| Distance to urban centres (URBAN_DIST) | Calculated from the centroid of the farm property to the nearest urban centre | Land and Information NZ (LINZ) https://data.linz.govt.nz/ |
| Distance to rivers (RIVER_DIST) | Calculated from the centroid of the farm to the nearest mapped river from vector line layer | Land and Information NZ (LINZ). https://data.linz.govt.nz/ |
| Social deprivation index (DEPRIV) | Extracted from raster layer by the intercept with the farm centroid. | Statistics NZ census data. Decile measure of socio-economic status; 1 being lowest and 10 highest. |
| Area of high quality pasture (HQ_HA) | Overlay of the vector polygon LCDB layer with farm property polygon layer to calculate number of hectares classified as ‘high producing exotic grassland’ | Land Cover Database (LCDB) Version 4. A satellite derived GIS vector polygon layer of land use. <https://lris.scinfo.org.nz> (1) |
| Area of low quality pasture (Hectares) (LQ_HA) | Overlay of the vector polygon LCDB layer with farm property polygon layer to calculate number of hectares classified as ‘low producing grassland’, ‘depleted grassland’, ‘tall tussock grassland’. | Land Cover Database (LCDB) Version 4. A satellite derived GIS vector polygon layer of land use. <https://lris.scinfo.org.nz> (1) |
| Forest cover (HA_FOREST) | The union of endemic and exotic forest cover of New Zealand as ha of each farm | <https://koordinates.com/layer/168-nz-native-polygons-topo-150k/>  <https://koordinates.com/layer/131-nz-exotic-polygons-topo-150k/> |
| Area of ‘other’ land cover (OTHER_AREA) | The size of the farm was calculated in post-GIS with the st_area command for the property polygon. HQP, LQP and FOR were subtracted from the total area to give this variable. | Agribase™ |
| Mean and standard deviation of annual temperature (TEMP) | The mean and sd of the annual temperature across the farm calculated from raster cells within a farm polygon | Land and Environments NZ (LENZ) layer (2). www.koordinates.com |
| Mean and standard deviation of mean minimum temperature of the coldest month (TEMP_MIN) | The mean and sd of mean minimum temperature of coldest month across the farm calculated from raster cells within a farm polygon | Land and Environments NZ (LENZ) layer (2). www.koordinates.com |
| Mean and standard deviation of annual solar radiation (SOLAR) | The mean and sd of the annual solar radiation across the farm calculated from raster cells within a farm polygon | Land and Environments NZ (LENZ) layer (2). www.koordinates.com |
| Mean and standard deviation of winter solar radiation (SOLAR_WINTER) | The mean and sd of the winter solar radiation across the farm calculated from raster cells within a farm polygon | Land and Environments NZ (LENZ) layer (2). www.koordinates.com |
| Annual water deficit (WATERDEF) | The mean and sd of the annual water deficit across the farm calculated from raster cells within a farm polygon. It is calculated from mean daily temperature, mean daily solar radiation, and mean monthly rainfall. | Land and Environments NZ (LENZ) layer (2). www.koordinates.com |
| Slope mean and standard deviation, aspect (SLOPE) | Slope x sin(aspect) and Slope x cosin(aspect) according to the methods in Stage (3) | Digital elevation model from Land and Information NZ (LINZ). https://data.linz.govt.nz/ |
| Elevation mean and standard deviation (DEM) | Mean and sd of farm elevation calculated from the grid falling within farm polygon | Digital elevation model from Land and Information NZ (LINZ). https://data.linz.govt.nz/ |
| Regional Council (COUNCIL NAME) | The Agribase farm polygon classified into one of the 17 regional council regions of NZ | NZ Regional Councils  <https://koordinates.com/layer/4240-nz-regional-councils-2012-yearly-pattern/> |

1. Thompson S, Gruner I, Gapare N. New Zealand Land Cover Database Version 2: illustrated Guide to Target Classes. In: Environment Mft, editor. Wellington, NZ2003.

2. Leathwick J, Morgan F, Wilson G, Rutledge D, McLeod M, Johnston K. Land Environments of NZ: A Technical Guide. Wellington, NZ: Ministry for the Environment; 2002.

3. Stage AR. An expression for the effect of aspect, slope, and habitat type on tree growth. Forest Science. 1976;22(4):457-60.
