# Supplementary material for: Species distribution models: A comparison of statistical approaches for livestock and disease epidemics
Source: PLoS One. 2017 Aug 24;12(8):e0183626. doi: 10.1371/journal.pone.0183626 (PMC5570337; doi:10.1371/journal.pone.0183626)
Supplement: S3 Table — The cattle prediction root mean squared error (RMSPE) using the withheld results for both individual farms within regions and to 3km quarantine zones. Table also shows the mean number of cattle per farm in the region, the standard deviation for all farms in brackets, and the total number of farms in each region. Results for Quarantine zone predictions are in 1,000s of cattle. (DOCX) [file pone.0183626.s003.docx]

**S3 Table: Model results for cattle.** *The cattle prediction root mean squared error (RMSPE) using the withheld results for both individual farms within regions and to 3km quarantine zones. Table also shows the mean number of cattle per farm in the region, the standard deviation for all farms in brackets, and the total number of farms in each region. Results for Quarantine zone predictions are in 1,000s of cattle.*

| **Region** | **Mean cattle per farm (SD)** | **Number of quarantine zones per region (10 simulations)** | **Total number of farms** | **RMSPE** | | | | | |
| --- | --- | --- | --- | --- | --- | --- | --- | --- | --- |
|  |  |  |  | **Farm-level RF** | **Farm-level BRT** | **Farm-level KNN** | **Quarantine zone RF** | **Quarantine zone BRT** | **Quarantine zone KNN** |
| **Overall** | **94.4 (243.1)** | **1997** | **91662** | **179.94** | **173.64** | **238.10** | **6.01** | **3.69** | **6.69** |
| Auckland | 27.7 (113.3) | 36 | 9815 | 91.76 | 93.13 | 141.10 | 1.00 | 1.50 | 2.18 |
| Bay of Plenty | 66.1 (197.5) | 59 | 5880 | 138.10 | 122.07 | 197.00 | 10.45 | 6.77 | 8.76 |
| Canterbury | 99.6 (345.6) | 455 | 12281 | 288.28 | 280.57 | 336.50 | 5.40 | 3.70 | 6.21 |
| Gisborne | 162.3 (407.7) | 65 | 1410 | 312.19 | 303.44 | 379.80 | 5.23 | 4.89 | 5.40 |
| Hawkes Bay | 104.0 (275.8) | 126 | 3972 | 190.20 | 202.43 | 280.30 | 5.89 | 3.21 | 3.68 |
| Manwt-Wngn | 104.0 (204.1) | 180 | 8759 | 130.61 | 123.53 | 207.40 | 3.13 | 1.82 | 3.53 |
| Marlborough | 37.8 (113.3) | 60 | 1900 | 90.47 | 109.54 | 172.60 | 2.09 | 2.74 | 3.43 |
| Nelson | 13.6 (45.4) | 2 | 195 | 30.27 | 34.59 | 107.50 | 0.11 | 0.54 | 5.87 |
| Northland | 91.3 (203.6) | 100 | 8385 | 119.13 | 110.05 | 181.60 | 10.92 | 2.10 | 12.61 |
| Otago | 88.9 (248.4) | 382 | 5038 | 197.99 | 197.12 | 304.90 | 3.12 | 3.09 | 4.20 |
| Southland | 132.2 (311.5) | 160 | 4859 | 255.13 | 247.18 | 315.10 | 3.20 | 3.12 | 3.51 |
| Taranaki | 124.3 (171.2) | 70 | 5294 | 108.35 | 102.86 | 165.40 | 6.22 | 4.21 | 5.45 |
| Tasman | 35.4 (130.7) | 26 | 2637 | 90.42 | 86.46 | 168.10 | 2.55 | 2.84 | 6.33 |
| Waikato | 138.4 (256.1) | 183 | 15473 | 180.22 | 164.10 | 227.00 | 11.17 | 6.25 | 12.78 |
| Wellington | 54.3 (151.9) | 59 | 4018 | 88.89 | 90.83 | 155.90 | 1.67 | 1.01 | 2.62 |
| West Coast | 103.3 (253.3) | 34 | 1746 | 178.58 | 179.40 | 226.00 | 6.72 | 2.02 | 9.41 |
