# Supplementary figures and images for: Species distribution models: A comparison of statistical approaches for livestock and disease epidemics
Source: PLoS One. 2017 Aug 24;12(8):e0183626. doi: 10.1371/journal.pone.0183626 (PMC5570337; doi:10.1371/journal.pone.0183626)

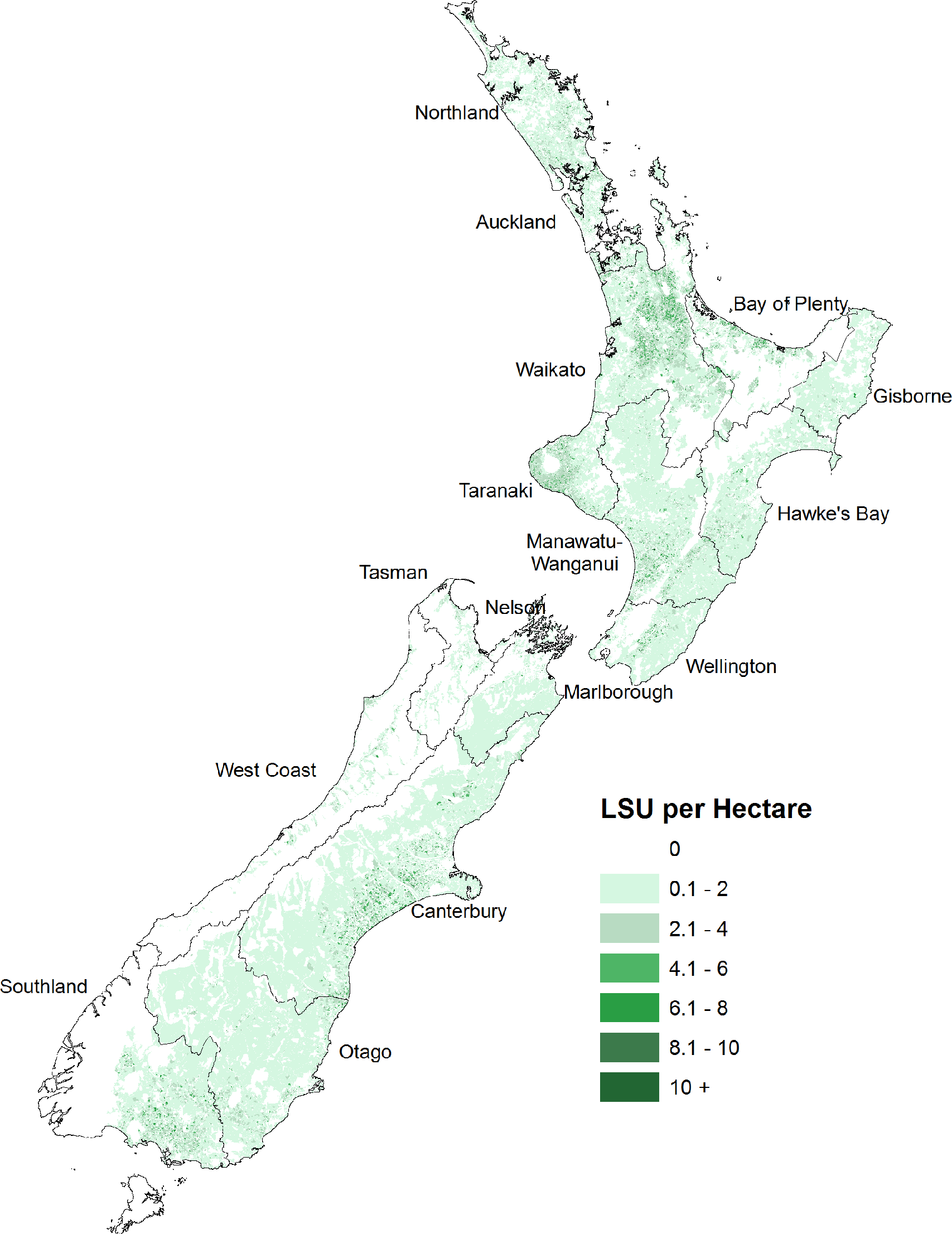

Supplement: S1 Fig — White areas represent areas removed such as cross leased land or areas of missing data. Areas of more than 10 LSU/cattle per hectare are represented in dark green. LSU represented per hectare is used for display purposes only. (TIF) [file pone.0183626.s004.tif]
